# Navigating the psychosocial landscape of Ehlers–Danlos syndrome: an autobiographic case study

**DOI:** 10.1007/s44192-025-00268-5

**Published:** 2025-08-17

**Authors:** Jessica D. Locke, Jason T. Eastman

**Affiliations:** 1 Independent Researcher, Myrtle Beach, SC USA; 2 Coastal Carolina University, Conway, SC USA

**Keywords:** Rare disorders, Ehlers–Danlos syndrome (EDS), Psychosocial impact, Chronic pain, Stigma and isolation

## Abstract

Living with a rare disorder, such as Ehlers–Danlos Syndrome (EDS), presents unique psychosocial challenges. This autobiographical case study explores the psychological, social, and professional impacts of navigating life with hypermobile EDS (hEDS), a condition marked by chronic pain, joint instability, and diagnostic uncertainty. Placing personal experiences into the context of clinical findings and research, this study highlights both the physical and emotional toll of the syndrome, including stigma, isolation, and medical gaslighting often accompanying rare conditions. The first-person case study of the first author extracted by the second author experienced in qualitative interviewing provides a first-hand account of the importance of addressing the psychosocial dimensions of rare disorders to foster understanding, empathy, and systemic improvements in patient care. In addition to calls for more encompassing medical care, this work also advocates for increased access to psychosocial support and recognition of the broader implications of living with rare, often invisible conditions.

## Introduction

While clinical perspectives often dominate discussions of rare diseases, the psychosocial implications—including identity formation, social relationships, and mental health—are critical because rare diseases and disabilities profoundly affect individuals’ psychosocial well-being and social participation. This study synthesizes findings from studies on rare diseases, disabilities, and their intersection with psychological and social dynamics into the first author’s experiences with Ehlers–Danlos Syndrome (EDS), a group of heritable connective tissue disorders characterized by joint hypermobility, skin hyperextensibility, and tissue fragility. The clinical presentation varies by subtype, with hypermobile Ehlers–Danlos Syndrome (hEDS) involving chronic pain and joint dislocations [22], while vascular Ehlers–Danlos Syndrome (vEDS) carries severe risks, such as arterial or organ ruptures.

Diagnosis relies on clinical criteria and genetic testing, though the genetic basis of some subtypes like hEDS remains unclear. Prevalence was traditionally estimated between 1 in 5000 to 20,000 individuals; however, due to its highly variable clinical presentation and frequent under recognition—particularly among males—this estimate likely underrepresents the true number of affected individuals [3]. A United Kingdom population-based study reported a much higher diagnosed prevalence of approximately 1 in 500 for hEDS and Hypermobility Spectrum Disorder (HSD) combined, highlighting the likelihood of widespread underdiagnosis [8].

This discrepancy in prevalence estimates carries important psychosocial implications, as delayed or missed diagnoses can lead to chronic frustration, depression, and reduced quality of life for affected individuals [25]. That is, due to their low prevalence and complex diagnostic processes, rare diseases present unique challenges. The “diagnostic odyssey” is a prominent feature of the condition, often accompanied by frustration, fear, and isolation among patients and families. Delayed diagnosis exacerbates these psychosocial complications as individuals experiencing diagnostic delays report higher levels of frustration, anxiety, and impaired social relationships [4]. While diagnostic clarity often alleviates uncertainty, it also introduces new psychological and social adjustments [2, 4].

Delayed diagnosis of physical health conditions, particularly rare diseases, exerts a profound psychological toll on affected individuals. Research indicates that patients who experience diagnostic delay report significantly higher levels of emotional distress, including frustration, irritability, anxiety, fear, and depressive symptoms. This emotional burden is often compounded by a lack of psychological care during the pre-diagnostic period, with only around a third of individuals receiving mental health support [4]. Moreover, delayed diagnosis is linked to various functional and social consequences, such as loss of independence, difficulty in maintaining employment or education, and challenges in explaining symptoms to others—factors that collectively contribute to social isolation and stigmatization. People describe a “narrative of chaos,” in which individuals feel powerless, unable to make future plans, and constantly face uncertainty regarding their health status. These psychosocial consequences underscore the urgent need for earlier recognition and diagnosis, along with integrated psychological support, to mitigate the emotional impact of prolonged diagnostic journeys [4].

### Personal background

Like many individuals with rare disorders, my diagnostic odyssey was long and filled with irritation, echoing research highlighting delays in diagnosis as a significant source of psychological distress. My journey with EDS began subtly, with symptoms that initially seemed unrelated and easily dismissed. As I moved through adolescence and adulthood, I noticed increasing issues with joint instability, chronic pain, and fatigue that persisted beyond what felt normal. Tasks others managed effortlessly like peeling potatoes often left me in pain, but without an apparent explanation I initially attributed my pain and frequent tendonitis to bad luck or overexertion. Repeated doctor visits often resulted in misdiagnoses or dismissals, leaving me feeling like my concerns were not valid. One example of feeling dismissed by doctors is hearing, “Well, your lab work is all normal,” after describing ongoing and debilitating symptoms. This can feel invalidating—like the doctor is implying that because nothing shows up on a test, your experience is not real or worth further investigation.

It was not until much later, after years of persistent symptoms and self-advocacy, that I was diagnosed with hEDS. I underwent ankle surgery in 2011, during which my surgeon noted the tendon appeared shredded, raising suspicion of an underlying connective tissue disorder. This observation prompted me to research conditions like Marfan syndrome and Ehlers–Danlos syndrome, where I recognized that many of my unexplained medical issues aligned more closely with EDS. In 2014, a geneticist at UCSF formally diagnosed me with Ehlers–Danlos syndrome, confirming my suspicions and providing clarity to years of unanswered questions.

Receiving the diagnosis of EDS elicited a profound sense of relief. After years of uncertainty and self-doubt, the confirmation offered emotional validation and alleviated the persistent fear my symptoms were imagined. It marked the first time I felt truly seen in a medical setting. Still, hEDS lacks a definitive genetic marker, and with this ambiguity came a mix of validation and uncertainty about how to move forward.

Since diagnosis, living with an invisible illness shaped many aspects of my life, introducing unique challenges in personal, social, and professional spaces. Explaining my limitations to others results in misunderstanding or skepticism, at times even accusations of making it up or exaggerating. I faced disbelief, stigma, and moments where others dismissed my pain or fatigue entirely. These experiences, while isolating, drove me to find my voice as an advocate for myself and others living with rare and invisible conditions.

## Management

hEDS management includes symptomatic care, physical therapy to improve joint stability and proprioception, and lifestyle modifications to prevent injuries. Additionally, managing comorbidities such as chronic fatigue, gastrointestinal issues, and autonomic dysfunction is essential to improve quality of life [14]. Multidisciplinary care is crucial for addressing the complex needs of EDS patients.

I focus on managing my symptoms through lifestyle adjustments, physical therapy, joint protection strategies, and working with a multidisciplinary team to reduce complications and maintain my daily functioning. Still, I face a constant internal debate about my physical limits. I constantly question whether I should stop doing something that could hurt me or whether I need to try harder and push through. For example, when it comes to activities like going to the gym, I often grapple with uncertainty: should I push myself harder to build strength and endurance, or will doing so increase my risk of injury? This creates a cycle of guilt and second-guessing: I feel like I failed if I choose to rest because I fear harming myself if I push too far; yet I feel self-disappointment if I do not push myself. I never really know where my limit lies; the uncertainty and anxiety exhaust me.

A sense of unpredictability also governs my life. On some days, I can manage activities that seem impossible for others; on other days it is impossible for me to manage activities others complete with ease. Planning or feeling confident in commitments is complicated because my body does not always cooperate. I avoid making plans in advance with others, knowing I might not feel up to it when the day comes—a difficult line to navigate between managing a chronic medical condition, persistent fatigue and pain, and the emotional weight of possible depression while knowing that one of the best prescriptions for depression is social interaction with others. In fact, I delayed plans made to finalize a draft of this very manuscript with the coauthor because of unforeseen physical complications.

### Medical care

Accessing specialized healthcare and genetic testing is a persistent challenge for individuals with rare disorders like EDS, especially hEDS which leaves patients in diagnostic limbo for years. The healthcare journey for someone with a rare disorder requires navigating fragmented systems not designed to address complex conditions. This journey often demands a high degree of self-advocacy, which can be exhausting and isolating. I often receive multiple declined referrals with the explanation that “we don’t treat this condition,” forcing me to begin the search for another provider and repeatedly contact my primary care office to submit yet another referral request. Over time, this process makes me feel like I am a burden to my primary care team—like I am annoying or a difficult patient simply for trying to access appropriate care.

Individuals living with rare disorders and chronic pain often face significant stigma when seeking relief through medications typically associated with substance use treatment. Patients with conditions like hEDS, who frequently experience nociplastic pain, are sometimes labeled as “drug-seeking” when conventional therapies fail. The use of medications like low-dose naltrexone (LDN), originally developed for substance abuse, can paradoxically increase that stigma—even though studies show it may be effective for pain reduction, mood stabilization, and energy enhancement at individualized doses [18].

As someone with hEDS, I experienced this firsthand. Despite the documented benefits of LDN for patients like me, including its effects on microglial activity and mast cell modulation, I encountered dismissive attitudes and skepticism from healthcare providers unfamiliar with its off-label use. This stigma not only delays treatment but also exacerbates psychological distress. When I secure much needed medicine, my friends and family question my use of it, asking whether I overdosed on my medications—particularly when I developed new symptoms clearly aligned with EDS but unfamiliar to them. This leaves me feeling devastated, hurt, and questioning myself; if even those closest to me do not believe what I am going through and my ability to manage it, why would I expect distant doctors to?

Ultimately, securing medical care means self-advocacy plays a critical role in managing EDS. My role as a self-advocate reflects the critical need for integrated care models to address the multifaceted impacts of rare disorders [1]. It involves educating healthcare providers about the condition, contesting insurance denials for necessary tests or treatments, and actively seeking out specialists familiar with rare diseases. However, the realities of this advocacy are daunting. For example, it often requires driving long distances to reach the few specialists knowledgeable about EDS. These trips frequently involve hours of travel, lengthy waiting periods, and significant out-of-pocket expenses, as not all specialists are in-network or covered by insurance. Such costs quickly accumulate, creating financial stress that compounds the emotional and physical toll of living with EDS.

The cost of non-covered alternative treatments—such as chiropractic care, massage therapy and maintaining a specialist-recommended diet—such as one free from dyes, preservatives, or inflammatory triggers—adds another layer of financial strain. While beneficial for symptom management, these dietary changes are often significantly more expensive than standard processed foods, further widening the gap between what is medically necessary and financially feasible. After recently calculating these additional living expenses related to EDS, I found they amount to approximately $500 per month. I often find myself with impossible choices: do I pay for a massage therapy session that keeps my pain manageable, or do I pay my electricity bill? I try to cut corners—buying cheaper foods or skipping chiropractic and massage therapy during difficult months—but my body cannot tolerate it. Each attempt to save money only leads to a sharp decline in my physical health, making the cycle even more overwhelming and emotionally draining.

The cumulative impact of these barriers underscores the urgent need for more expansive, yet integrated care models that streamline access to specialists, provide comprehensive psychosocial support, and minimize the financial burden on patients. Peer-reviewed research emphasizes the importance of such models for improving outcomes in rare disease management [1]. Until these systems are implemented, the burden of navigating and funding care disproportionately falls on patients, leaving them to manage their condition and the complex logistics of accessing appropriate care.

## Psychosocial aspects & quality of life

Living with EDS or any rare disorder introduces unique psychosocial challenges that extend far beyond the physical symptoms. Furthermore, life is incredibly challenging for individuals like myself with coexisting conditions such as premenstrual dysphoric disorder (PMDD), attention-deficit/hyperactivity disorder (ADHD), and spatial and auditory processing disorders in addition to Dysautonomia, Mast Cell Activation Disorder, and possible periodic paralysis, which are often secondary due to EDS. These conditions compound the difficulties in understanding and managing EDS, creating a sense of being overwhelmed and misunderstood. Research indicates that individuals with rare diseases experience heightened levels of anxiety and depression due to the prolonged search for answers and the dismissive attitudes they often encounter from healthcare providers [20]. I felt this deeply myself and after years of unexplained symptoms, I began to question my own reality. At times, I wondered whether I overreacted or imagined things—despite knowing my body was not functioning the way it should.

Internal conflict, fueled by medical gaslighting which is defined as an invalidation of patient concerns without proper evaluation [17], took a significant toll on my mental health and eroded my trust in the healthcare system. In fact, some researchers go as far as describing medical gaslighting as trauma inducing [23]. As a woman, I am more likely to experience medical gaslighting than male patients [13]. At the same time, given my own experiences working in the medical field I also understand arguments that gaslighting partially stems from doctor’s time constraints and pressures for productivity confound their efforts to engage in empathetic communication and patient-centered care [11].

Beyond the healthcare system, living with chronic pain and fatigue presents its own set of psychological challenges. Research shows that chronic pain often correlates with higher risks of anxiety and depression, particularly when individuals feel powerless over their condition [27]. These findings resonate deeply with my experience of navigating both physical and emotional struggles with EDS. Fatigue further exacerbates the psychological strain, contributing to feelings of inadequacy and self-doubt. Isolation is another prevalent theme, as peers and even healthcare providers struggle to comprehend the full impact of an invisible illness. This lack of understanding often leaves individuals feeling disconnected and unsupported, further compounding their emotional distress.

### Social dynamics

Rare diseases significantly reduce social participation. Factors such as unemployment, mobility limitations, and poor access to support systems intensify social isolation [5, 19]. Employment and personal well-being are crucial determinants of social inclusion, underscoring the need for targeted interventions. My transition from being “able-bodied” to living with a chronic illness like Ehlers–Danlos Syndrome (EDS) is particularly challenging when one’s career is tightly intertwined with identity and purpose. Working as a paramedic represented a job and a calling—demanding physical resilience, mental acuity, and the ability to endure high-stress situations. I once prided myself on these attributes, but ultimately made the very emotional realization that my body could no longer sustain the demands of position. This set me back not just practically and financially; it undermined my sense of purpose and self-worth.

I experienced a profound disconnect between my medical training and my self-perception as a patient. Even as a trained medical professional, I often found myself dismissing my symptoms, doubting their validity, or minimizing their impact. I wrestled with taking myself seriously, a challenge many patients face but one I paradoxically experienced despite knowing better. This duality—a paramedic who doubted her own medical needs—was deeply unsettling, highlighting the stigma and internalized shame that often accompany invisible illnesses.

However, experiencing the patient side of the healthcare system gave me invaluable perspective. It equipped me with an understanding many medical professionals lack, allowing me to empathize more deeply with patients and navigate my care more effectively. My medical knowledge helped me identify and articulate my symptoms more clearly, advocate for appropriate interventions, and make informed decisions about my treatment. While this unique position has not erased the challenges, it provided me tools to manage them more effectively and a renewed sense of purpose in advocating for others with similar struggles.

The decision to pursue an advanced degree in psychology reflects my efforts to redefine my identity to accommodate my physical limitations while allowing me to continue helping others. This shift represents both resilience and adaptability, traits often developed through navigating chronic illness. Research supports the importance of vocational and educational adjustments in improving the quality of life for individuals with chronic conditions [24]. By channeling my experiences into new opportunities, I found new ways to honor the aspects of my identity that matter most—compassion, service, and the drive to make a meaningful impact—while acknowledging the realities of my condition.

### Interpersonal relationships

Living with EDS has been, and still is a profoundly complex and emotional journey that affects not just myself, but those around me and my relationships with them. I often feel ashamed of having a rare disorder, as though my condition is something I need to hide or apologize for. This feeling stems from societal expectations to appear “normal” and my internal pressure to not seem weak or different. I sometimes find myself reluctant to share my struggles, fearing judgment or pity, which only deepens the isolation that comes with living with an invisible illness. Yet at the same time, failure to disclose my conditions to others strains my relationships with them given the unpredictable ebbs and flows of my symptoms.

Everyday situations also reveal how misunderstood invisible disabilities like EDS often are, even among close family members. During a recent city tour with my brother, we ended up arguing over where to park. He preferred finding free parking further away, while I insisted on paying for a spot closer to our destination. To him, walking an extra half a mile seemed insignificant—but for me, that distance could mean the difference between enjoying the outing or triggering pain and fatigue lasting for days. He could not understand why such a seemingly small detail mattered so much. Moments like these highlight the ongoing emotional management explain and justify my limitations, even to those who care about me.

Living with EDS significantly shapes my social relationships because I often fear being perceived as a burden or being rejected by friends due to my limitations. Activities that might seem trivial or routine for others, such as going on a roller coaster or participating in group sports, are often impossible for me. These moments of exclusion—deliberate or incidental—leave me feeling left out or alienated. I worry that my inability to participate fully will make me less desirable as a friend, leading to anxiety about whether my condition will drive people away. My feelings of exclusion from social activities mirror studies revealing reveal how rare diseases limit participation and intensify isolation [19] along with the need for improved accessibility and awareness to foster inclusion [2].

One of the more subtle yet deeply challenging aspects of living with a rare and invisible condition is the internal struggle to determine what qualifies as advocacy versus what could be perceived as whining, complaining, or seeking attention. It is a fine line I often find myself navigating with uncertainty. I want to raise awareness, to express my needs clearly, and to speak up when something is not right—but I also worry about how I others perceive me. Will I be dismissed as dramatic or a hypochondriac? Am I saying too much, or not enough? This constant self-monitoring adds a layer of emotional fatigue, making it difficult to confidently assert myself without questioning the validity or tone of my own voice.

### Family dynamics

Family dynamics add another layer of genetic complexity to highly emotional social relationships. I come from a small rural town in Germany with limited genetic diversity. Growing up, I heard stories of family members and neighbors with “weak joints,” “bad skin,” or other unexplained health problems that, looking back now, fit the picture of a hereditary connective tissue disorder that may have persisted in my family for generations, even if it was never recognized as such. While there may be a family history of related symptoms—such as joint pain or fatigue—there is often a lack of formal diagnoses or shared understanding. This absence of validation can make finding solace or guidance in familial relationships difficult. Encouragement to “try harder” or “toughen up” during childhood reflects a cultural tendency to minimize invisible conditions, unintentionally fostering feelings of inadequacy and guilt in those affected.

Managing my hEDS within family settings continues to be a challenge. Rare disorders like mine require continuous education and advocacy, but not every family member is open to understanding or fully acknowledging the complexities of my condition. I sometimes feel compelled to defend the legitimacy of my experience, which can strain relationships and create emotional distance. Even within my closest supports, I feel isolated at times—like in reading about the psychosocial impact of hypermobile hEDS in families, I could not help but recognize many of the same patterns in my own experience.

De Percin et al. [9] describe how families affected by EDS often experience a mix of emotions—relief when a diagnosis finally validates their struggles, but also frustration, fear about the future, and tension between family members who understand the condition differently. They describe how some relatives may cope by withdrawing or avoiding activities, while others may push through until burnout or injury. Their findings also highlight how relationships can become strained when the invisible nature of the condition is not fully understood by others.

I personally lived through many of these dynamics, both within my immediate family and across generations. While I am the only one in my family who has received a formal diagnosis of hEDS, I strongly believe I am not the only one affected. Living in different countries with different healthcare systems makes it difficult for my family members to access the same diagnostic resources available to me in the United States. However, several of my relatives, both direct and extended, experience symptoms or have been diagnosed with other conditions that strongly suggest EDS may run in our family. Chronic joint issues, unexplained pain, easy bruising, fatigue, and other connective tissue-related problems are common on both sides of my family, even when these symptoms were never given a name.

Documenting these experiences is deeply personal, but I feel it is important. My story is not just about my own diagnosis—it is about recognizing a wider family pattern going unnoticed for too long. It is about the emotional weight of carrying a condition that many of my relatives likely share but may never have had the chance to understand. I hope that by sharing not only my journey but also the larger family context, those closest to me will see how deeply psychosocial factors—such as medical uncertainty, distance, and lack of awareness—shape the experience of living with a rare and often invisible condition like hEDS.

### Disability stigma and social identity

The social identity approach to disability emphasizes the interplay between individual and societal perceptions. According to Dirth and Branscombe [10], disability is not solely a medical issue but a socio-political construct shaped by cultural and structural factors. This aligns with the biopsychosocial model, which integrates biological, psychological, and social dimensions of disability [2].

Disabilities often result in societal stigma, marginalizing individuals and limiting their access to resources. Individuals often face stigma and disbelief, even from medical professionals, as their symptoms are invisible or poorly understood. Medical gaslighting—where their experiences are invalidated or dismissed—compounds the difficulty of seeking care. The “invisible disability” label fosters isolation, as others may fail to recognize the legitimacy of the struggles associated with conditions like EDS. Fear of exacerbating symptoms, shame surrounding perceived physical limitations, and the emotional toll of chronic pain further deepen the psychological burden. Even children with disabilities, for example, encounter exclusion and negative perceptions from peers. Early interventions focusing on inclusive education and positive exposure to disabilities can mitigate these biases [2].

I personally felt the impact of this stigma in my relationships—one of which ended with the painful remark, “When I met you, we hiked a mountain in Colorado, and now I had to push you in a wheelchair through an airport.” Comments like this reveal how quickly support can turn into judgment when disability becomes visible, and how profoundly misunderstood the progression of chronic illness can be.

### Mental health

Mental health challenges are prevalent among individuals with disabilities and rare diseases. Depression and anxiety are common, often exacerbated by negative social interactions and limited support networks [25]. Psychological distress associated with adjusting to physical disabilities highlights the importance of tailored psychological interventions [21]. A nationwide Swedish study confirmed individuals with EDS have heightened risk for bipolar disorder, ADHD, depression and attempted suicide although it is unclear if the associations are due to genetics or the environment—which likely includes the distress causes by having and managing the condition [7]. Eccles and Owens [12] propose the high co-occurrence of joint hypermobility and autonomic hyperactivity may be particularly relevant to neurodevelopmental profiles, noting overlapping features between hEDS and ADHD such as heightened sensory sensitivity, anxiety, and difficulty with focus and organization. Understanding these connections provides important context for the psychosocial experiences of patients with hEDS, whose symptoms may be compounded by neurodevelopmental traits that impact daily functioning, coping strategies, and interactions with the healthcare system.

Personally, before I even received a diagnosis, I struggled with constant feelings of frustration, isolation and a deep sense of uncertainty; after finally receiving a diagnosis of hEDS, I experienced an emotional cascade that was both validating and disorienting. On one hand, the clarity offered immense relief after years of uncertainty; on the other, it surfaced a profound sense of grief for the life I had unknowingly adapted around invisible symptoms. The diagnosis marked the beginning of a psychological reckoning, as I started to process decades of medical gaslighting, isolation, and misunderstood pain. I sought mental health support to navigate the emotional aftermath, including anxiety and a lingering fear of not being believed.

In retrospect, the link between hEDS and neurodevelopmental conditions like ADHD became increasingly apparent, with growing scientific recognition of their comorbidity [4]. Recent research sheds light on the neurodevelopmental and psychiatric dimensions of hypermobility conditions. Casanova et al. (2020) conducted a comprehensive review of existing literature and clinical data, concluding that individuals with hEDS and hypermobility spectrum disorders (HSD) exhibit significantly higher rates of neurodevelopmental conditions such as ADHD and autism spectrum disorder, as well as psychiatric issues including anxiety, depression, and mood instability [2].

These findings support the growing recognition that the impact of hypermobility is not limited to connective tissue symptoms, but also extends to the brain and nervous system. For patients, this means that psychological symptoms are not merely secondary to chronic pain or disability, but may instead represent core features of the condition itself—a realization that can offer powerful validation and reshape how support is approached clinically and socially. In my case, I was also diagnosed with ADHD, which further aligned with these findings and helped explain longstanding cognitive and emotional challenges that had previously been overlooked or misattributed. This overlap brought new understanding to lifelong struggles with executive dysfunction and sensory overwhelm, deepening my commitment to psychosocial healing as much as physical symptom management. Fortunately, resilience is possible often with some of the same coping mechanisms that alleviate the physical symptoms of my hEDS.

## Coping mechanisms and resilience

Research suggests tailored psychosocial and physical interventions significantly enhance the quality of life for individuals with chronic conditions [24]. Cognitive-behavioural techniques and Acceptance and Commitment Therapy (ACT) show promise in managing chronic symptoms by targeting maladaptive thought patterns, increasing psychological flexibility, and improving overall functioning in individuals with persistent pain and complex conditions [15, 16, 26]. These approaches are particularly valuable in populations with rare or poorly understood disorders, where symptom unpredictability and diagnostic uncertainty often contribute to emotional distress and decreased quality of life.

Over time, I discovered strategies that work for my mind as well as my body. For me, yoga and meditation along with massage therapy offer physical relief and emotional grounding. These practices let me move freely and build strength without putting pressure on my joints, which is essential for someone with my physical needs and limitations. When I am suspended in the air, there is a feeling of weightlessness and freedom that goes beyond just physical exercise—it is empowering. I am keenly aware that yoga is not for everyone. It must be approached carefully, especially by those with joint issues, as overdoing it or practicing without proper guidance can lead to injuries. I also made meditation an integral part of my routine. It provides a space to breathe, center myself, and calm the mental chaos that can accompany chronic health challenges. Even just a few minutes a day helps me find clarity and peace, giving me greater control over my emotions and responses to stress. It is a simple practice that cultivated resilience and mindfulness. Massage therapy is another essential part of my care because it prevents muscle spasms, eases tension, and improves my mobility. It is not just a treat but a necessity for keeping my body functional and comfortable. After a session, I often feel physically and mentally rejuvenated, as if some of my weight has been lifted.

I also sometimes practice Reiki, an energy healing technique. While I am unsure whether the benefits are due to proven energy work or simply a placebo effect from telling my brain it will help, it brings a sense of calm and comfort. Whether it is a mind–body connection or something more, the important part is that I feel better after practicing it. There is value in anything that helps us feel supported, even if the mechanism behind it is not entirely clear.

These practices—yoga, meditation, massage therapy, Reiki along with a natural diet—became part of a deeply personal and individualized approach to my well-being. I am always mindful that every person is different, and what helps me might not resonate with someone else. Finding what works takes time and self-awareness, and no one-size-fits-all solution exists. These practices remind me that I possess the tools to care for myself and can create moments of peace, strength, and hope even in the face of challenges.

## Strategies for psychosocial support

Support networks, including family, peer groups, and patient associations, are critical in mitigating psychological distress. Access to credible information and community support improves coping mechanisms and quality of life [4, 25]. The dynamics of my condition taught me the importance of seeking connections with others who truly understand what living with a rare disorder entails. I found solace in communities of individuals with similar conditions and in forging relationships with those who value and accept me for who I am, not for what I can or cannot do.

In an effort to foster community and reduce isolation among individuals facing similar challenges, I founded a local support group for people with EDS and other rare disorders. This group became a valuable space for sharing experiences, coping strategies, and emotional support. It has been incredibly meaningful to connect with others who truly understand the daily struggles of living with a rare condition—something that is often difficult for others to fully grasp. Meeting people with the same issues helped normalize my experiences and reinforced the importance of peer support in managing chronic, often misunderstood conditions. Still, as described above, my fear of rejection and longing for deeper understanding within social and family circles remain constant undercurrents.

## Discussion

The psychosocial impact of living with a rare disorder is profound, often shaped by medical uncertainty, social isolation, and emotional distress along with the physical symptoms. The diagnostic odyssey, medical gaslighting, and fragmented care systems exacerbate feelings of frustration, anxiety, and even loss of self-identity. Beyond the physical challenges, individuals with rare disorders frequently encounter stigma, skepticism, and inadequate support networks, which can lead to significant mental health struggles such as depression, anxiety, and trauma responses. These realities highlight the need to move beyond a symptom-centric approach to care and embrace a biopsychosocial model that acknowledges the intersection of physical, emotional, and social well-being [4, 10, 19].

Medical management of rare disorders, while essential for symptom control and physical health, often fails to account for the profound psychosocial effects of living with a chronic condition. I often share personal moments of frustration—not because I am looking for sympathy, but because as a former paramedic I lived and worked in a broken healthcare system long enough to see how it wears people down. From medical professionals afraid to step outside rigid protocols, to patients like me left to manage complex conditions on our own, the system simply isn’t built for those who don’t fit the standard mold. And here’s the thing—I can’t even be mad at the doctors because of the way the system is built. It is set up to protect itself, not us. We live in a country where doctors get reprimanded for thinking outside the box, so it is understandable they stick to the textbook, even when the textbook does not fit someone like me.

Despite these systemic challenges, resilience strategies empower individuals to navigate life with a rare disorder. Psychosocial support, peer networks, advocacy efforts, and self-education emerged as critical tools for fostering resilience. Many individuals find strength in connecting with others who share similar experiences, engaging in patient-led advocacy, and utilizing mental health interventions tailored to medical trauma and chronic illness. Cognitive reframing, acceptance-based approaches, and lifestyle adaptations are vital coping mechanisms. By fostering self-advocacy and emotional resilience, individuals can reclaim a sense of agency over their condition and life trajectory.

Yet, resilience should not be an individual burden—it must be collectively supported through systemic change. There is a critical need for increased awareness, research, and institutional support to bridge the existing healthcare and social inclusion gaps. Psychosocial well-being is not a secondary concern but a critical component of comprehensive care. Anxiety, depression, and trauma responses often stem from medical experiences rather than the disease itself, exacerbating physical symptoms through stress-induced flares. Research highlights the bidirectional relationship between mental health and disease management, where chronic stress can heighten pain perception, worsen autonomic dysfunction, and contribute to maladaptive coping strategies [4].

Thus, integrating mental health care and social support into routine medical management is beneficial and necessary for improving long-term outcomes [2]. Medical professionals must receive better training to recognize and validate rare conditions, while policy initiatives should prioritize funding for rare disease research, patient advocacy programs, and accessible healthcare services. Community education is equally vital in reducing stigma and fostering inclusive environments that empower individuals rather than isolate them [25].

Holistic care acknowledges that medical treatment alone cannot ensure a patient’s well-being. A multidisciplinary approach—including mental health professionals, occupational therapists, and patient advocacy networks—can provide patients with the tools to navigate their condition’s physiological and emotional aspects. Therapy for processing medical trauma, cognitive-behavioral techniques for managing chronic pain, and support groups for sharing experiences all contribute to improved coping mechanisms and resilience. Community-based interventions, such as peer mentorship programs, adaptive work environments, and structured patient-led initiatives, foster a sense of belonging and purpose. This enhances emotional stability and empowers individuals to advocate for better medical care and societal accommodations.

A medical paradigm shift is needed to progress in rare disease care. One that views psychosocial health not as an adjunct to physical treatment but as an integral pillar of disease management is needed. By incorporating mental health and social support structures into standard care models, we move closer to providing patients with the comprehensive, dignified, and effective care they deserve. Effectively addressing the psychosocial aspects of rare diseases demands a fundamental shift in perspective—one that goes beyond a purely medical focus to acknowledge patients as whole individuals with diverse needs and meaningful contributions. Through sustained advocacy, multidisciplinary collaboration, and patient-centered research, we can cultivate a future where individuals with rare disorders are not just treated—but truly understood, supported, and empowered to thrive.

## Data Availability

The authors confirm that all autobiographical data generated or analyzed during this study are included in this published article. As the only subject of the study, the first author offers to make themselves available for reader inquiries should they arise.
